# High-pressure microwave-assisted pretreatment of softwood, hardwood and non-wood biomass using different solvents in the production of cellulosic ethanol

**DOI:** 10.1186/s13068-023-02272-9

**Published:** 2023-02-07

**Authors:** Dawid Mikulski, Grzegorz Kłosowski

**Affiliations:** Faculty of Natural Science, Department of Biotechnology, Kazimierz Wielki University, Ul. K. J. Poniatowskiego 12, 85-671 Bydgoszcz, Poland

**Keywords:** Bioethanol, Biomass, High-pressure microwave-assisted pretreatment, HG technology

## Abstract

**Background:**

Pretreatment is an indispensable stage of the preparation of lignocellulosic biomass with key significance for the effectiveness of hydrolysis and the efficiency of the production of cellulosic ethanol. A significant increase in the susceptibility of the raw material to further degradation can be attained as a result of effective delignification in high-pressure conditions. With this in mind, a method of high-pressure pretreatment using microwave radiation and various solvents (water, 40% *w*/*v* NaCS, 1% *v*/*v* H_2_SO_4_, 1% *w*/*v* NaOH or 60% *v*/*v* EtOH with an addition of 1% *v*/*v* H_2_SO_4_) was developed, enabling the acquisition of biomass with an increased susceptibility to the process of enzymatic hydrolysis. The medium obtained in this way can be used for the production of cellulosic ethanol via high-gravity technology (lignocellulosic media containing from 15 to 20% dry weight of biomass). For every type of biomass (pine chips, beech chips and wheat straw), a solvent was selected to be used during the pretreatment, guaranteeing the acquisition of a medium highly susceptible to the process of enzymatic hydrolysis.

**Results:**

The highest efficiency of the hydrolysis of biomass, amounting to 71.14 ± 0.97% (glucose concentration 109.26 ± 3.49 g/L) was achieved for wheat straw subjected to microwave-assisted pretreatment using 40% *w*/*v* NaCS. Fermentation of this medium produced ethanol concentration at the level of 53.84 ± 1.25 g/L. A slightly lower effectiveness of enzymatic hydrolysis (62.21 ± 0.62%) was achieved after high-pressure microwave-assisted pretreatment of beech chips using 1% *w*/*v* NaOH. The hydrolysate contained glucose in the concentration of 91.78 ± 1.91 g/L, and the acquired concentration of ethanol after fermentation amounted to 49.07 ± 2.06 g/L. In the case of pine chips, the most effective delignification was achieved using 60% v/v EtOH with the addition of 1% *v*/*v* H_2_SO_4_, but after enzymatic hydrolysis, the concentration of glucose in hydrolysate was lower than in the other raw materials and amounted to 39.15 ± 1.62 g/L (the concentration of ethanol after fermentation was ca. 19.67 ± 0.98 g/L). The presence of xylose and galactose was also determined in the obtained fermentation media. The highest initial concentration of these carbohydrates (21.39 ± 1.44 g/L) was observed in beech chips media after microwave-assisted pretreatment using NaOH. The use of wheat straw after pretreatment using EtOH with an addition of 1% *v*/*v* H_2_SO_4_ for the preparation of fermentation medium, results in the generation of the initial concentration of galactose and xylose at the level of 19.03 ± 0.38 g/L.

**Conclusion:**

The achieved results indicate a high effectiveness of the enzymatic hydrolysis of the biomass subjected to high-pressure microwave-assisted pretreatment. The final effect depends on the combined use of correctly selected solvents for the different sources of lignocellulosic biomass. On the basis of the achieved results, we can say that the presented method indicates a very high potential in the area of its use for the production of cellulosic ethanol involving high-gravity technology.

## Background

Bioethanol is the most frequently manufactured biofuel from plant biomass. In its dehydrated form, it makes an independent fuel or is an additive to petroleum fuels. The global production of ethanol fuel in 2021 amounted to 27 billion gallons (according to a report by the RFA—Renewable Fuels Association), with the biggest manufacturers of bioethanol being the United States (15 billion gallons), which use corn as raw material, and Brazil (7.5 billion gallons) producing ethanol from sugarcane. However, in view of the rising demand for ethanol as a fuel, a cheap, easily available and renewable raw material, which is not used for the production of food and feed, is necessary for its manufacture [1]. Lignocellulosic biomass, which can be successfully used as a source of carbon in biosynthesis processes, is a raw material meeting the above requirements. The key factor limiting its widespread use in the production of bioethanol is its resilient, complex structure. It is a building material rather than a reserve material like starch, and the combined presence of cellulose, hemicellulose and lignins determines its high resistance to biodegradation. In order to increase the susceptibility of lignocellulosic biomass to enzymatic degradation, an additional stage is introduced to the process of the production of cellulosic bioethanol—it is referred to as pretreatment [2, 3]. Its task is to increase the share of the amorphous areas in cellulose and a degradation of hemicellulose, as well as a removal of lignins [4, 5]. There are many different types of pretreatment including fragmentation, the use of ultrasounds, diluted and concentrated inorganic acids, alkali and organic solvents, ionic liquids, surfactants, deep eutectic solvents, steam explosion, ammonia (AFEX) and many more [6–10]. The majority of pretreatment methods are carried out in the conditions of an increased pressure and temperature, which facilitates the process of delignification and the partial decomposition of structural polysaccharides. Unfortunately, such conditions may facilitate processes of the dehydration of monosaccharides, leading to the generation of pretreatment by-products, i.e. furfural from xylose or 5-(hydroxymethyl) furfural (5-HMF) from glucose. These compounds are undesirable in larger concentrations since they are considered fermentation inhibitors negatively affecting the activity of yeast [11, 12]. In addition, the intense thermobaric processing makes the process more cost-intensive. This problem can be solved, however, through the use of microwave radiation marked by a higher energy efficiency in comparison to conventional heating. Advantages of the use of microwaves also include the possibility of the immediate cessation of heating; direct heating of the entire volume of the biomass rather than through the walls of the container in which it is kept or through heat exchangers; the degradation of hydrogen bonds through electromagnetic radiation, and a considerable rate of the heating of the biomass [13, 14]. In practice, in order to increase the effectiveness of pretreatment using microwave radiation, it is used in combination with various solutions such as diluted organic or inorganic acids, alkali, ionic liquids, deep eutectic solvents, organic solvents or ammonia [15–20]. The use of microwave radiation for the pretreatment of corn residues increases the susceptibility of the biomass to enzymatic hydrolysis even by ca. 15% in comparison to the conventionally heated biomass [21]. In addition, the biomass from wheat and rye stillage after microwave-assisted pretreatment using diluted sulfuric acid shows a higher susceptibility to enzymatic hydrolysis in comparison to conventionally heated biomass, which manifests itself in the acquisition of a higher concentration of glucose by respectively 13 and 30 mg/g DW [22, 23]. Microwave radiation in an alkaline environment (microwave-assisted alkali) is also a more effective way of the pretreatment of rice straw biomass in comparison to the alkaline pretreatment using ultrasound (ultrasound-assisted alkali) and ball milling pretreatment. Hydrolysates after the enzymatic degradation of rice straw subjected to microwave-assisted pretreatment yielded 2 g/L more glucose than the biomass after ultrasound-assisted pretreatment and 4 g/L more glucose than the biomass after ball milling pretreatment [24].

An effective pretreatment of lignocellulosic biomass determines a high susceptibility of structural polysaccharides to enzymatic hydrolysis, although it does not guarantee a high concentration of sugars in the fermentation medium. An economically sound production of cellulosic ethanol can take place only in process conditions guaranteeing the acquisition of a high concentration of ethanol in a fermentation medium at the level of 40 g/L, which necessitates the lowering of the cost of distillation to a rational level. One solution to the problem can lie in the use of HG (high gravity) technology, as a part of which lignocellulosic media containing from 15 to 20% DW of biomass are used. The use of HG technology requires, however, that enzymatic hydrolysis results in the acquisition of glucose concentration in the fermentation medium at the level of at least 80 g/L, which in turn is possible only after effective pretreatment [25]. Differences in the structure of softwood, hardwood and non-wood biomasses determine the suitability of the individual methods of pretreatment, which calls for their careful choice. An effective use of different sources of lignocellulose in the production of ethanol involving HG technology requires a precise determination of the suitability of the particular substances such as diluted acids, alkali or organic solvents for microwave-assisted biomass pretreatment. This work presents effects of the use of various solvents, i.e. water, 1% *v*/*v* sulfuric acid solution, 1% *w*/*v* sodium hydroxide solution, 40% *w*/*v* sodium cumene sulfonate solution (NaCS), and 60% *v*/*v* ethanol solution with the addition of 1% *v*/*v* H_2_SO_4_ combined with high-pressure microwave-assisted pretreatment on the effectiveness of the enzymatic hydrolysis of pine chip (softwood), beech chip (hardwood) and wheat straw (non-wood) biomasses. The paper discusses the impact of the applied process parameters on the generation of by-products of pretreatment. It also presents data on the course of fermentation and the achieved yield of cellulosic ethanol using the HG technology. Aspects of scientific novelty are complemented with a presentation of very detailed results of analyses of the biomass obtained as a result of high-pressure microwave-assisted pretreatment covering the use of such techniques as XRD, FTIR and SEM, identification of changes in the biomass structure and the suitability of biomass for the production of cellulosic ethanol. The results of tests of the pretreatment of different types of biomass performed in the environment of high pressure generated using microwaves, under changeable environmental conditions and involving different solvents, examining their suitability for efficient production of cellulosic ethanol, have not yet been published. What is a novelty here is the results of the tests of the pretreatment of different types of biomass performed under high pressure generated using microwaves, in changeable environmental conditions and involving different solvents, in order to check their suitability for the efficient production of cellulosic ethanol. The generated high yield of ethanol per tonne of raw material constitutes an area of significant progress in the improvement of the effectiveness of the use of lignocellulosic biomass for the production of bioethanol. So far, only conventional heating was used in studies testing high-pressure technology in the production of cellulosic ethanol [26, 27]. We would also like to point out the fact that the development of the technology of production of ethanol from lignocellulosic biomass is gradual and it is possible to assume from publications in this scope that we are dealing here with the deepening of knowledge and the upgrading of the existing technology.

## Results and discussion

### Influence of high-pressure microwave-assisted pretreatment on the composition of lignocellulose

High-pressure microwave-assisted pretreatment was performed for three types of biomass: pine chips (softwood), beech chips (hardwood), and wheat straw (non-wood) under constant pressure of 156 PSI using different solvents (water, 40% *w*/*v* NaCS, 1% *v*/*v* H_2_SO_4_, 1% *w*/*v* NaOH or 60% *v*/*v* ethanol with the addition of 1% *v*/*v* H_2_SO_4_). The application of a high pressure with 1% *v*/*v* H_2_SO_4_ led to the charring of all biomass types and made further analyses for this type of pretreatment impossible. In turn, the use of the other solvents enabled an extraction of biomass components, including hydrophobic compounds, i.e. lignins (Table 1). All the analysed types of lignocellulosic biomass after microwave-assisted pretreatment using water were marked by the lowest content of cellulose and the highest content of hemicellulose and lignins. Also the level of extractives of different types of biomass after pretreatment using water was the lowest (Table 1). In view of the above, the biomass samples after microwave-assisted pretreatment using water were treated as reference samples and the results generated using the other variants of pretreatment with other solvents were compared to them.

**Table 1:** Characteristics of different types of lignocellulosic biomass after high-pressure pretreatment

Pretreatment conditions	Type of biomass	Type of solvent used for the pretreatment	Biomass extractives (%)	Lignocellulose composition (% DW)			Biomass crystallinity (%)	Hydrolysis susceptibility of biomass* [mg/g DW]	Hydrolysis yield* [%]
				Cellulose	Hemicellulose	Lignin			
156 PSI, 60 min., 600 W	Pine chips	H_2_O	28.68 a ± 1.54	57.05 a ± 0.15	4.65 ac ± 1.40	36.00 a ± 0.00	55.93	96.31 a ± 1.25	15.20 a ± 0.20
		40% w/v NaCS	46.37 b ± 0.05	72.81 b ± 0.18	2.33 b ± 0.76	22.74 b ± 0.04	66.26	152.47 b ± 0.78	18.85 b ± 0.10
		1% w/v NaOH	39.44 c ± 3.42	65.47 c ± 0.03	3.43 bc ± 1.06	28.64 c ± 0.54	69.03	115.38 c ± 2.13	15.86 a ± 0.29
		60% v/v EtOH + 1% v/v H_2_SO_4_	44.75 bc ± 3.81	72.11 b ± 0.31	2.56 b ± 0.64	21.54 d ± 0.14	66.77	244.69 d ± 3.88	30.54 c ± 0.49
	Beech chips	H_2_O	31.32 a ± 0.01	66.00 c ± 0.90	4.86 ac ± 1.25	16.65 e ± 0.35	65.96	352.07 e ± 0.44	48.01 d ± 0.06
		40% w/v NaCS	54.59 d ± 1.04	88.46 d ± 0.36	2.02 b ± 0.80	6.15 f ± 0.35	72.93	533.97 f ± 4.47	54.33 e ± 0.45
		1% w/v NaOH	47.34 b ± 2.30	83.00 e ± 0.40	4.47 ac ± 0.35	6.65 f ± 0.35	73.24	573.61 g ± 5.66	62.21 f ± 0.62
		60% v/v EtOH + 1% v/v H_2_SO_4_	59.51 d ± 2.24	84.02 e ± 0.50	2.43 b ± 0.35	7.09 f ± 0.10	70.64	530.56 f ± 4.69	56.84 g ± 0.50
	Wheat straw	H_2_O	42.47 bc ± 1.25	62.45 f ± 0.35	6.86 d ± 0.55	20.05 g ± 0.05	60.98	455.01 h ± 0.88	65.58 h ± 0.13
		40% w/v NaCS	58.14 d ± 1.67	86.40 g ± 0.10	4.30 ac ± 0.66	4.00 h ± 0.10	66.10	682.85 i ± 9.29	71.14 i 0.97
		1% w/v NaOH	56.32 d ± 1.18	83.36 e ± 0.54	5.34 d ± 0.64	1.60 i ± 0.10	68.58	1.48 j ± 0.22	0.16 j ± 0.02
		60% v/v EtOH + 1% v/v H_2_SO_4_	47.93 b ± 1.00	76.65 h ± 0.95	5.56 d ± 0.35	4.40 h ± 0.60	64.24	530.88 f ± 1.13	62.34 f ± 0.13

^*^after 72 h of hydrolysis under the following conditions: pH 5.5, 16 FPU/g DW, 160 g DW/L. The mean values given in columns within the table with different letter indices are significantly different (α < 0.05). *DW* dry weight

High-pressure microwave-assisted pretreatment of pine chips using 40% *w*/*v* NaCS or 60% *v*/*v* EtOH (with an addition of H_2_SO_4_) made it possible to obtain biomass marked by the highest content of cellulose (ca. 72% DW) and the lowest content of lignins (ca. 21.5–22.5% DW). In such process conditions also the highest level of extraction of biomass components was achieved at ca. 44.5–46.5% (Table 1). The use of 40% *w*/*v* NaCS or 60% *v*/*v* EtOH (with an addition of H_2_SO_4_) in microwave-assisted pretreatment of pine chips enabled a reduction of the lignin content by ca. 39%. The process of the delignification of beech chips in microwave conditions had a similar effectiveness using 40% *w*/*v* NaCS, 1% *w*/*v* NaOH and 60% *v*/*v* EtOH (with an addition of H_2_SO_4_), and made it possible to reduce the lignin content by ca. 60%. The use of the above-mentioned solvents during high-pressure pretreatment made it possible to acquire biomass with a higher content of cellulose at the level of even 88.46 ± 0.36% for the biomass after pretreatment using 40% w/v NaCS (Table 1). It turned out that the most effective way of the delignification of wheat straw was pretreatment using 1% w/v NaOH, which enabled a reduction of lignin content by as much as over 90%. Noteworthy was also a high degree of reduction of the content of lignins generated using the 40% solution of *w*/*v* NaCS, which reached the level of 4.00 ± 0.10% DW, with the highest level of extraction of biomass components of more than 58% (Table 1). High-pressure microwave-assisted pretreatment enables the acquisition of lignocellulosic biomass of a different origin in which cellulose is the dominating component and the content of lignins and hemicellulose is strongly reduced. Our own earlier research has already proved the suitability of microwave-assisted hydrotropic pretreatment for the delignification of beech chip (a reduction by ca. 20%) and wheat straw (a reduction by ca. 45%) biomasses, but the results presented herein indicate that the application of a higher pressure significantly intensifies the process of the lignin removal [28]. Microwave-assisted pretreatment was conducted for 60 min, because in our earlier study delignification of different types of lignocellulosic biomass was conducted using microwaves and NaCS for 15, 30 and 60 min. The extension of the duration of pretreatment from 15 to 60 min at 117 PSI resulted in an increase in the level of delignification, which increased the reduction of the content of lignins from 12.21 to 36.58% in pine chips biomass, from 22.49 to 57.68% in beech chips biomass and from 25.43 to 74.08% in wheat straw biomass. The coefficient of regression of the growth of the level of reduction of the lignin content over time in lignocellulosic biomass of different origin ranged from 0.8485 to 0.9679 [28]. Other authors have indicated that pretreatment using microwave radiation and 0.75% w/v NaOH leads to a reduction of lignin content by ca.10% in the biomass of catalpa sawdust [29]. In turn, the use of a 60% v/v ethanol solution combined with an exposure of sawdust mixture to microwave radiation makes it possible to reduce lignin content by ca. 40% [30]. The effectiveness of NaCS in the delignification of rice straw at an increased temperature in comparison to another hydrotrope (Na-X, sodium xylene sulfonate) has also been proved by other authors. A 20% solution of NaCS enabled an extraction of ca. 31% of components of rice straw biomass at 121 °C—the temperature was generated in a conventional way. Our own tests revealed a similar or a higher level of the removal of biomass components using water as a solvent in the conditions of microwave radiation (Table 1). This indicates a high usefulness of microwave-assisted pretreatment in the preparation of both biomass for enzymatic decomposition and hydrolysates for biosynthesis processes. The NaCS solution was also a more effective solvent, delignifying rice straw by ca. 51%, which facilitated the acquisition of a higher cellulose content in the biomass. Also our own tests proved that a removal of hydrophobic substances, i.e. lignins, from the biomass, increases the content of its cellulose (Table 1) [31]. The effectiveness of microwave radiation in the delignification of eucalyptus biomass was demonstrated using ionic liquids as a solvent. The use of microwave radiation made it possible to reduce the lignin content by ca. 63%, which was ca. 40% more than in the case of conventional heating. Similarly to our own tests, an increase in the content of cellulose in the eucalyptus biomass was also determined after the removal of lignins using microwaves and ionic liquids in comparison to the native biomass [32].

### Formation of by-products during high-pressure microwave-assisted pretreatment

The presence of a higher concentration of by-products of pretreatment in the fermentation medium may be a key parameter having an impact on the assessment of its suitability for fermentation in view of its direct effect on the fermentation activity of yeast [33]. The intensity of the generation of fermentation inhibitors during pretreatment has a smaller influence on the effectiveness of the process of fermentation in the case of the use of biomass separated from the solution after pretreatment. However, a qualitative and quantitative assessment of the presence of by-products is one of the elements marking a given method of preparation of the raw material for enzymatic hydrolysis and indicates whether it is necessary to detoxify the hydrolysate in order to perform an effective fermentation [34]. In the present study, it was discovered that regardless of the biomass type, the use of a 40% *w*/*v* NaCS solution and a 60% *v*/*v* EtOH acid solution in combination with high-pressure microwave-assisted pretreatment led to the generation of the highest concentrations of products of the dehydration of sugars (5-HMF and furfural) as well as phenolic compounds constituting products of lignin fermentation (Table 2). The use of the 60% v/v EtOH acid solution facilitates an intensive dehydration of xylose, which results in a high content of furfural; the highest concentration of the latter—in excess of 6200 mg/L—was discovered in the medium from wheat straw. The second product of the dehydration of sugars, i.e. 5-HMF being a product of the dehydration of glucose, is intensively generated in the environment of 40% *w*/*v* NaCS (Table 2). A 60% *v*/*v* EtOH acid solution facilitates the extraction of sinapyl alcohol, as manifested by the presence of this compound in concentrations of as much as in excess of 5.200 mg/L when pine or beech chips are used. Independently of the raw material used, only the presence of products of the fermentation of lignins, i.e. vanillic acid, sinapyl alcohol, guaiacol and syringaldehyde was discovered in all the compounds after pretreatment obtained when using 1% *w*/*v* NaOH and intensive microwave heating (Table 2). Analyses indicate a relation between the presence of pretreatment by-products and the effectiveness of the delignification process. An increased concentration of products from the dehydration of sugars, mainly 5-HMF, in hydrolysates from corn residues biomass was discovered after microwave-assisted pretreatment. In comparison to conventional heating, an increase in the concentration of 5-HMF by as much as up to ca. 1 g/L of hydrolysate from corn residues was observed after microwave-assisted pretreatment [21]. Other authors’ studies confirm our own observations indicating a relationship between the presence of pretreatment by-products and the type and conditions of the pretreatment and biomass type. Our own test as described herein revealed a higher concentration of products from the dehydration of sugars, i.e. furfural and 5-HMF, in solutions after pretreatment in an acid medium (EtOH with 1% v/v H_2_SO_4_). The increased generation of these compounds was determined as a result of the pretreatment of corn stover biomass using acids [35–37]. In turn, the presence of a heightened concentration of products from the degradation of lignins, i.e. vanillin, vanillic acid, 4-hydroxybenzaldehyde and syringaldehyde, was discovered in the hydrolysate from corn residues after pretreatment using wet oxidation [35]. A heightened concentration of phenolic compounds was also determined in wheat straw and pine foliage hydrolysates after pretreatment using wet oxidation [35, 38].

**Table 2:** Concentration of by-products in solutions after high-pressure pretreatment of different types of biomass

Pretreatment conditions	Type of biomass	Type of solvent used for the pretreatment	Concentration of by-products in the solution after biomass pretreatment [mg/L]								
			5-HMF	Furfural	1,2-dihydrobenzene	4-hydroxybenzoic acid	Vanillic acid	Sinapyl alcohol	Guaiacol	Syringaldehyde	Furoic acid
156 PSI, 60 min., 600 W	Pine chips	H_2_O	< LOD	< LOD	0.00 ± 0.00	0.00 ± 0.00	8.79 a ± 2.17	0.00 ± 0.00	0.00 ± 0.00	5.38 a ± 0.51	0.00 ± 0.00
		40% w/v NaCS	4162.15 a ± 7.88	1340.06 a ± 4.13	0.00 ± 0.00	0.00 ± 0.00	185.72 b ± 0.17	3.01 a ± 0.60	9.62 ac ± 0.11	55.71 b ± 0.33	< LOD
		1% w/v NaOH	< LOD	0.00 ± 0.00	0.00 ± 0.00	0.00 ± 0.00	1.87 a ± 0.14	2.96 a ± 0.61	63.53 b ± 1.87	72.32 c ± 2.51	0.00 ± 0.00
		60% v/v EtOH + 1% v/v H_2_SO_4_	623.10 b ± 13.05	1553.63 b ± 2.65	0.00 ± 0.00	0.00 ± 0.00	232.63 c ± 7.61	5274.31 b ± 6.32	14.01 c ± 0.07	40.47 d ± 0.40	< LOD
	Beech chips	H_2_O	< LOD	< LOD	0.00 ± 0.00	0.00 ± 0.00	9.79 a ± 0.20	0.00 ± 0.00	0.00 ± 0.00	< LOD	0.00 ± 0.00
		40% w/v NaCS	961.83 c ± 2.12	3475.10 c ± 5.73	53.62 a ± 0.41	23.90 a ± 0.44	411.34 d ± 2.44	132.54 c ± 0.04	57.50 d ± 0.77	20.39 e ± 0.06	< LOD
		1% w/v NaOH	0.00 ± 0.00	0.00 ± 0.00	0.00 ± 0.00	< LOD	< LOD	131.95 c ± 7.83	60.69 bd ± 2.63	30.35 f ± 1.85	24.31 a ± 1.37
		60% v/v EtOH + 1% v/v H_2_SO_4_	433.41 d ± 18.33	5945.93 d ± 9.97	7.15 b ± 0.54	2.55 b ± 0.39	394.84 e ± 3.56	5251.73 b ± 5.53	12.61 c ± 0.31	10.99 g ± 0.96	0.46 b ± 0.10
	Wheat straw	H_2_O	294.00 e ± 20.25	1089.59 e ± 0.33	0.00 ± 0.00	< LOD	103.17 f ± 4.65	0.00 ± 0.00	7.38 a ± 0.04	19.36 e ± 0.52	4.71 c ± 0.11
		40% w/v NaCS	1344.61 f ± 43.44	1223.45 f ± 07.49	91.49 c ± 1.00	24.04 a ± 0.74	210.67 g ± 0.77	65.04 d ± 1.85	42.44 e ± 1.14	33.93 h ± 1.89	< LOD
		1% w/v NaOH	0.00 ± 0.00	< LOD	0.00 ± 0.00	< LOD	5.21 a ± 0.02	90.22 e ± 1.80	72.59 f ± 1.49	36.58 h ± 0.84	112.68 d ± 1.69
		60% v/v EtOH + 1% v/v H_2_SO_4_	51.47 g ± 0.30	6289.71 g ± 7.50	0.00 ± 0.00	< LOD	163.85 h ± 0.72	3525.08 f ± 17.72	21.75 g ± 0.17	18.97 e ± 0.38	0.00 ± 0.00

The mean values given in columns within the table with different letter indices are significantly different (α < 0.05). Limit of detection (LOD): 5-HMF < 10.40 mg/L, furfural < 24.75 mg/L, 1,2-dihydrobenzene < 0.78 mg/L, 4-hydroxybenzoic acid < 0.42, vanillic acid < 0.34 mg/L, sinapyl alcohol < 0.97 mg/L, guaiacol < 1.55 mg/L, syringaldehyde < 3.54 mg/L, furoic acid < 0.22 mg/L

### Changes in the structure of lignocellulose as a result of high-pressure microwave-assisted pretreatment

To characterise changes in the structure of lignocellulose being an effect of the use of the presented pretreatment method, an analysis of the crystallinity of the biomass, FTIR and SEM were performed. As a result of microwave-assisted pretreatment under high-pressure involving 40% *w*/*v* NaCS, 1% *w*/*v* NaOH or 60% *v*/*v* ethanol with an addition of 1% *v*/*v* H_2_SO_4_, a lowered content of amorphous substances such as lignins or hemicellulose was observed, which resulted in an increase in the biomass crystallinity (Table 1). The highest increase in the level of biomass crystallinity was observed in samples of plant material after pretreatment using 1% *w*/*v* NaOH. In comparison to the biomass after pretreatment using water, an increase in the degree of the crystallinity of biomass after using 1% *w*/*v* NaOH amounted to ca. 13% for pretreatment using water, and ca. 7.5% for beech chips and wheat straw. Despite a high level of extraction of lignins and a high content of cellulose in all the types of biomass after microwave-assisted pretreatment using 40% *w*/*v* NaCS, the lowest increase in the crystallinity of biomass was recorded in comparison to the pretreatment using water (Table 1). The generated results indicate a probability of the presence of cellulose in an amorphous form in the indicated biomass types. An increase in the crystallinity of biomass as a result of microwave-assisted pretreatment using hot water, alkalis or ionic liquids was also observed by other authors. They discovered an increase in the crystallinity of bamboo biomass from 49.5 to 56.9% as a result of microwave-assisted pretreatment using hot water [39], an increase in the crystallinity of catalpa sawdust biomass from 32.3 to 39.4% as a result of pretreatment using microwaves and NaOH [29], and an increase in the crystallinity of eucalyptus biomass from 55.0 to 59.5% after microwave-assisted pretreatment using ionic liquids [32]. The use of surfactants to increase the solubility of hydrophobic substances, i.e. lignins, during pretreatment causes changes to the crystallinity of pine foliage biomass. A reduction of the content of lignins in the biomass even by ca. 73% decreases the share of amorphous substances and increases the crystallinity of the biomass. Just like in the analysed biomass types, an increase in biomass crystallinity from 32.7% to 48.5% was discovered in the pine foliage biomass after pretreatment [40].

**Fig.1:**
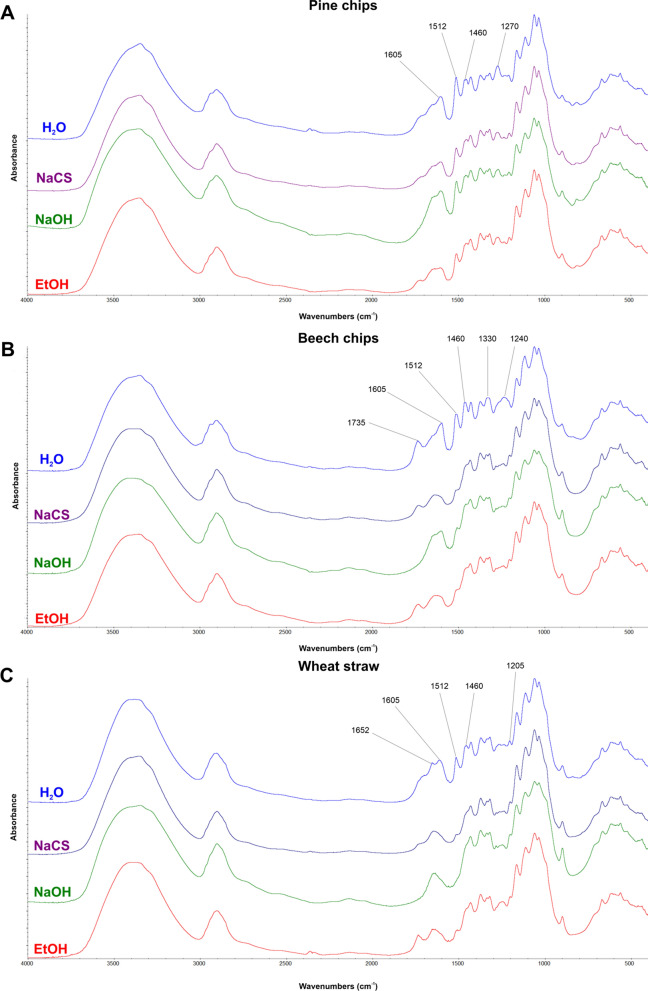
FTIR spectra of pine chips (**A**), beech chips (**B**) and wheat straw (**C**) after high-pressure pretreatment using different solvents

The effectiveness of microwave-assisted pretreatment in the conditions of high pressure using different solvents was also confirmed with the help of FTIR analyses (Fig. 1.). Absorbance bands of the analysed biomass types, related to the structure of lignins, i.e. 1230–1270, 1460, 1512 and 1605 cm^−1^, were weakened as a result of structural changes resulting from microwave-assisted pretreatment in the conditions of very high pressure [41]. As a result of the pretreatment of pine chips using 40% *w*/*v* NaCS and 60% *v*/*v* EtOH (with the addition of H_2_SO_4_), the region at wavenumber 1512 cm^−1^ attributed to the vibration of lignin aromatic rings was less intense. Pretreatment of pine chips generated changes manifesting themselves in the weakening of the absorbance band 1270 cm^−1^ attributed to guaiacyl subunits (guaiacyl lignin), characteristic for lignins originating from softwood biomass, which are constructed from coniferyl alcohol (guaiacyl lignin, G-lignin) [42]. An analysis of the beech chip biomass subjected to microwave-assisted pretreatment using 40% *w*/*v* NaCS, 1% *w*/*v* NaOH or 60% *v*/*v* EtOH with an addition of 1% *v*/*v* H_2_SO_4_ revealed changes marked by weakened absorbance bands at 1240, 1330, 1460, 1512 and 1605 cm^−1^ corresponding to structures characteristic for lignins. A decreased intensity of the spectrum at 1240 cm^−1^ is a characteristic feature of hardwood biomass and corresponds to syringyl ring stretching [32]. What is also interesting is the weakening of the intensity of the peak at 1735 cm^−1^ characteristic for hemicellulose, mainly acetate groups observed as a result of an analysis of beech chip biomass after pretreatment using NaCS and NaOH [41]. In the wheat straw biomass after pretreatment using NaCS, NaOH and EtOH, the highest weakening was recorded for bands at 1512 and 1605 cm^−1^ corresponding to the vibration of lignin aromatic rings [43].

**Fig.2:**
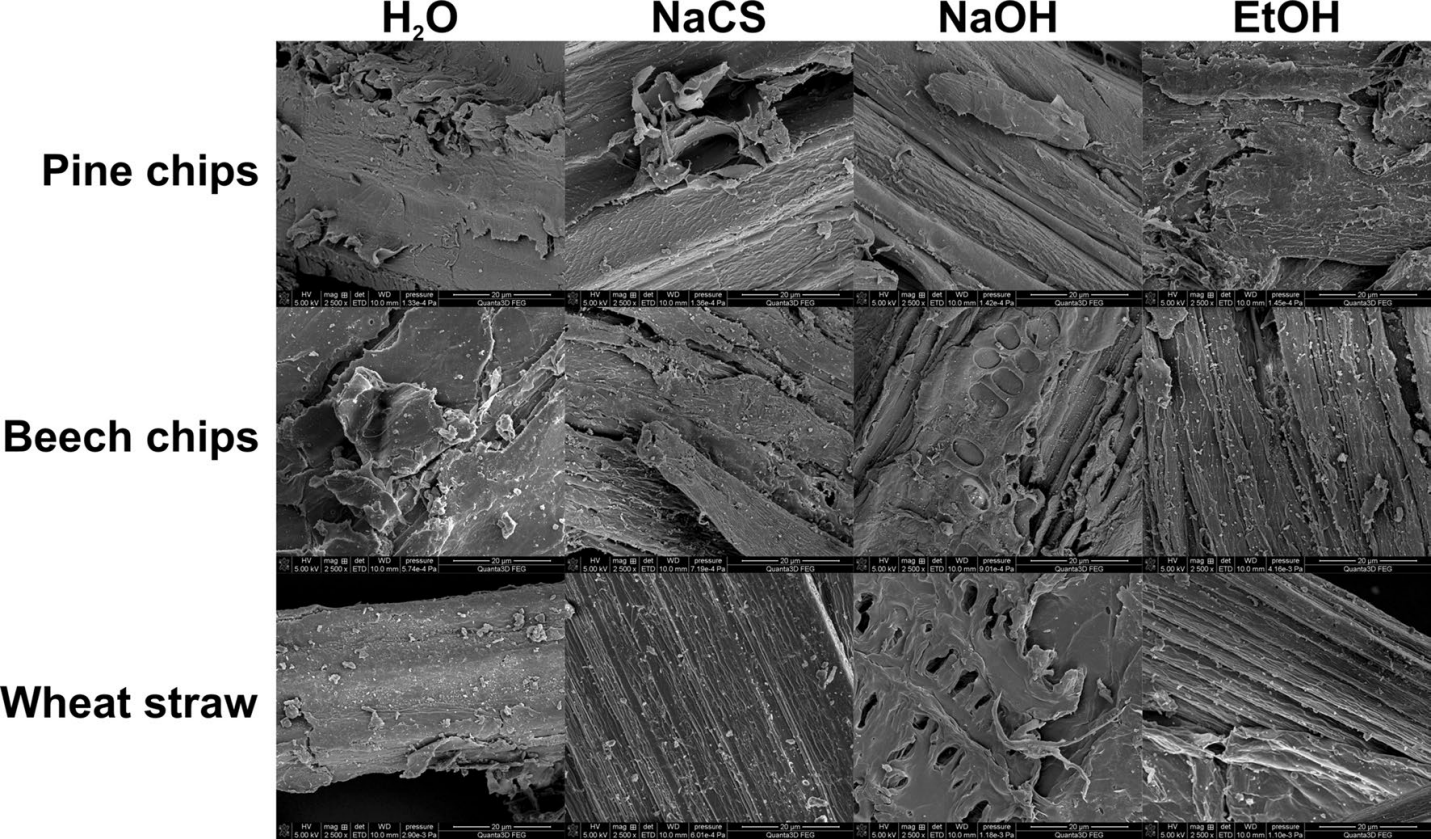
SEM images of pine chip, beech chip and wheat straw biomass after high-pressure pretreatment using different solvents

In order to more comprehensively document changes in the structure of biomass after microwave-assisted pretreatment under high pressure, imaging was performed with the help of a scanning electron microscope (SEM). Biomass obtained in such conditions using water as solvent was marked by smoothness and high orderliness (Fig. 2.). As a result of the use of NaCS, NaOH and EtOH during pretreatment, the surface of the biomass undergoes changes, becoming more porous and fibrous, which is conducive to the process of enzymatic hydrolysis by increasing the surface of exposure to cellulolytic enzymes [44]. Beech chip and wheat straw biomasses even show openings generated as a result of microwave-assisted pretreatment using NaOH (Fig. 2.). Similar observations were made for wheat straw biomass after pretreatment using hot water and sulfuric acid [45]. The emergence of openings as a result of pretreatment using NaOH was also confirmed for pine wood biomass [46].

### Susceptibility of biomass after high-pressure microwave-assisted pretreatment to enzymatic hydrolysis

The process of enzymatic hydrolysis was conducted for every type of biomass after microwave-assisted pretreatment in the conditions of high pressure using 40% *w*/*v* NaCS, 1% w/v NaOH or 60% *v*/*v* EtOH with an addition of 1% *v*/*v* H_2_SO_4_. Enzymatic hydrolysis was performed at pH 5.5, 50 °C, with an enzyme dose of 16 FPU/g DW of biomass and the biomass concentration of 160 g/L for 72 h. On the basis of the quantity of the released glucose in the solution, the hydrolysis susceptibility of biomass expressed in mg/g DW of biomass and the hydrolysis yield taking into account the quantity of biomass used and the content of cellulose in the biomass were calculated. The use of 40% *w*/*v* NaCS, 1% *w*/*v* NaOH or 60% *v*/*v* EtOH with an addition of 1% *v*/*v* H_2_SO_4_ during high-pressure pretreatment causes an increase in the susceptibility of biomass to hydrolysis and the yield of hydrolysis in comparison to biomass after pretreatment using water. From amongst the raw materials under analysis, softwood biomass (pine chips) is marked by the lowest susceptibility to hydrolysis and the lowest hydrolysis yield (Table 1). The maximum quantity of glucose released from this raw material amounts to ca. 245 mg/g DW, and the yield of hydrolysis was ca. 30% after pretreatment using 60% *v*/*v* EtOH with an addition of 1% *v*/*v* H_2_SO_4_. Despite the high resistance of pine chip biomass to enzymatic hydrolysis, the use of high-pressure pretreatment in the environment of microwaves together with EtOH makes it possible to double the yield of enzymatic hydrolysis in comparison to the biomass after pretreatment using water (Table 1). Beech chip biomass is marked by a heightened susceptibility to enzymatic hydrolysis in comparison to softwood biomass, which makes it possible to acquire the maximum quantity of glucose at the level of more than 570 mg/g DW and a hydrolysis yield of ca. 62% in the case of biomass after pretreatment using 1% *w*/*v* NaOH. The highest susceptibility to hydrolysis using cellulases was discovered for wheat straw. The applied conditions of enzymatic hydrolysis made it possible to generate ca. 683 mg glucose per gram of biomass and a hydrolysis yield at the level of 71% from the non-wood biomass after pretreatment using 40% w/v NaCS (Table 1). In contrast to other types of biomass, wheat straw after pretreatment using 1% *w*/*v* of NaOH solution was entirely resistant to the action of enzymes from the group of cellulases. A very important achievement of this stage of research was the selection, for each type of biomass, of the most effective solvent to be used during high-pressure pretreatment, allowing to generate a high concentration of glucose as a result of enzymatic hydrolysis. The use of a 60% *v*/*v* EtOH solution with an addition of sulfuric acid for the preparation of pine chips, 1% *w*/*v* NaOH for the preparation of beech chip biomass, and 40% *w*/*v* NaCS for the preparation of wheat straw was a way of pretreatment guaranteeing the generation of biomass highly susceptible to hydrolysis. Because of differences in the structure of softwood, hardwood and non-wood lignocellulose, it is necessary to approach the issue of the selection of the pretreatment method individually, which requires detailed analyses of the particular raw materials. An assessment of the effectiveness of the use of different solvents during microwave-assisted pretreatment of various types of biomass is very significant, as it constitutes a key stage of the construction of the process of bioconversion into ethanol, deciding about the ultimate productivity of the entire process. The obtained results allow us to conclude that the suitability of the particular solvents for the pretreatment of biomass depends on individual features of each raw material. The above is signalled in other publications. For instance, it was indicated that cassava has a higher susceptibility to enzymatic hydrolysis after microwave-assisted pretreatment involving a solution of NaOH than after one using H_2_SO_4_ [47]. Differences in the suitability of individual solvents for the pretreatment of lignocellulose concern not only different types of the raw material, but even a particular variety within a single species. It was confirmed that in the case of hemp biomass, the variety Eletta Campana is highly susceptible to hydrolysis after pretreatment using acid or alkali, whilst other varieties under analyses guaranteed the achievement of the highest concentration of glucose only after pretreatment using NaOH [48]. Other authors’ studies demonstrated a higher susceptibility of cellulose in rice straw biomass to enzymatic hydrolysis after pretreatment using NaCS at 121 °C. As a result of enzymatic hydrolysis using cellulases, ca. 500 mg of glucose was generated per gram of rice straw biomass, which is a poorer result in comparison to our own results for beech chips biomass after microwave-assisted pretreatment using 40% *w*/*v* NaCS, 1% *w*/*v* NaOH, 60% *v*/*v* EtOH + 1% *v*/*v* H_2_SO_4_, and for wheat straw biomass after pretreatment using 40% *w*/*v* NaCS (Table 1) [31]. A lower effectiveness of the hydrolysis of biomass in comparison to our own tests under description was also achieved using eucalyptus biomass after pretreatment using microwaves and ionic liquids. The maximum concentration of glucose generated for eucalyptus biomass amounted to ca. 410 mg/g [32]. Also the yield of glucose obtained as a result of the hydrolysis of cashew apple bagasse after alkaline microwave-assisted pretreatment was lower than that generated as a part of our tests and amounted to the maximum of ca. 375 mg/g of biomass using the enzyme dose at the level of 15 FPU/g DW over 72 h of hydrolysis [49]. The biomass generated as a part of our own tests, highly susceptible to enzymatic hydrolysis, indicates the usefulness of the suggested method of pretreatment for the process of the production of cellulosic ethanol. Even the use of a 10-times higher dose of cellulolytic enzymes (200 FPU/g DW) in the pretreatment of catalpa sawdust biomass after alkaline microwave-assisted pretreatment did not allow to generate a concentration of glucose in excess of 300 mg/g of biomass [29]. A high level of the delignification of pine foliage biomass generated as a result of pretreatment using surfactants made it possible to obtain a concentration of glucose similar to our own tests at the level of ca. 590 mg/g DW. However, the highest glucose yield was generated when the dose of cellulolytic enzyme was much higher, i.e. at the level of 100 FPU during 96 h of the process of hydrolysis [40].

### Kinetics of alcoholic fermentation of medium obtained from biomass after high-pressure microwave-assisted pretreatment

**Fig.3:**
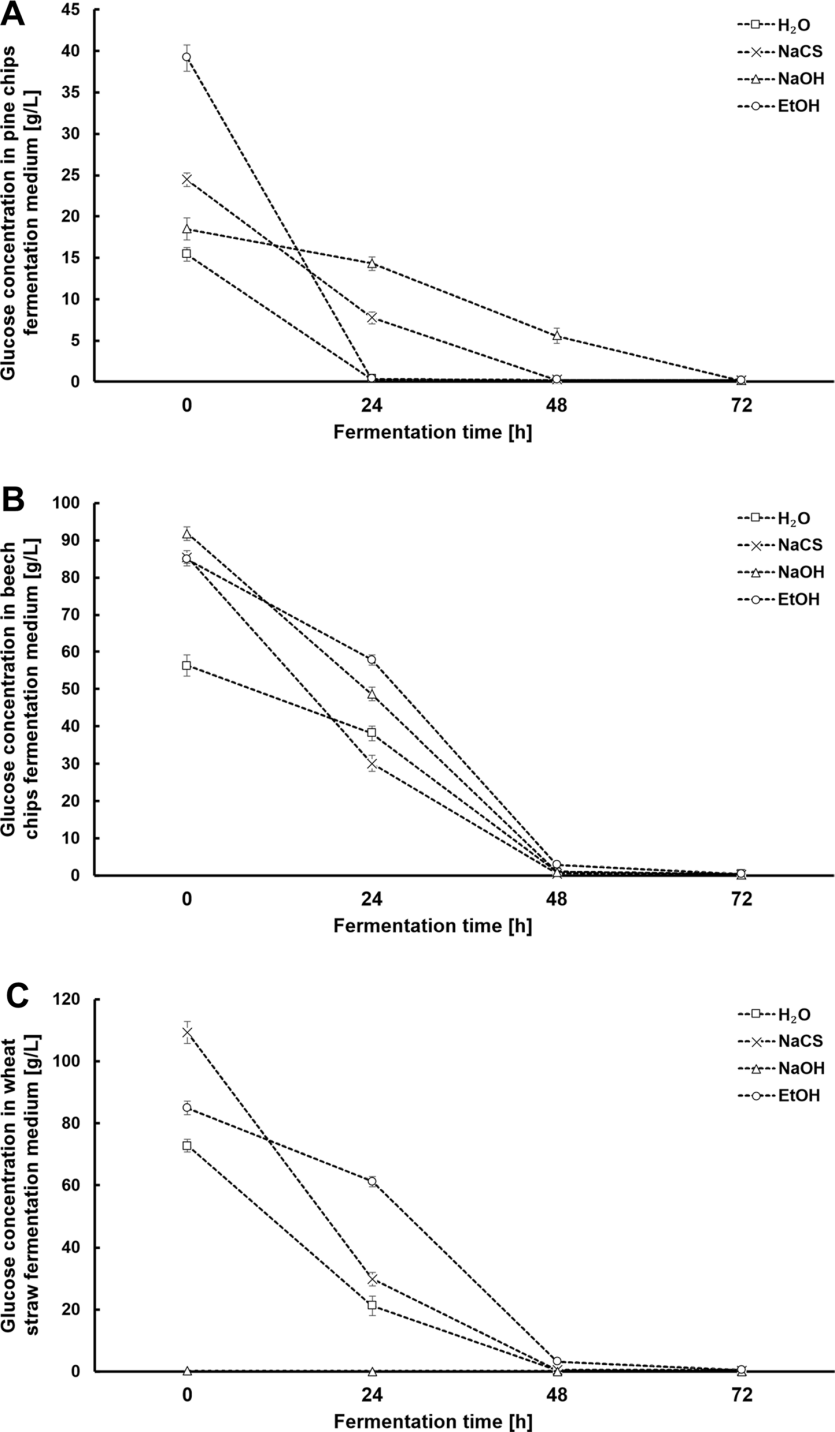
Changes in the concentration of glucose during alcoholic fermentation in hydrolysates obtained from pine chip (**A**), beech chip (**B**) and wheat straw (**C**) biomasses after high-pressure pretreatment using different solvents

**Fig.4:**
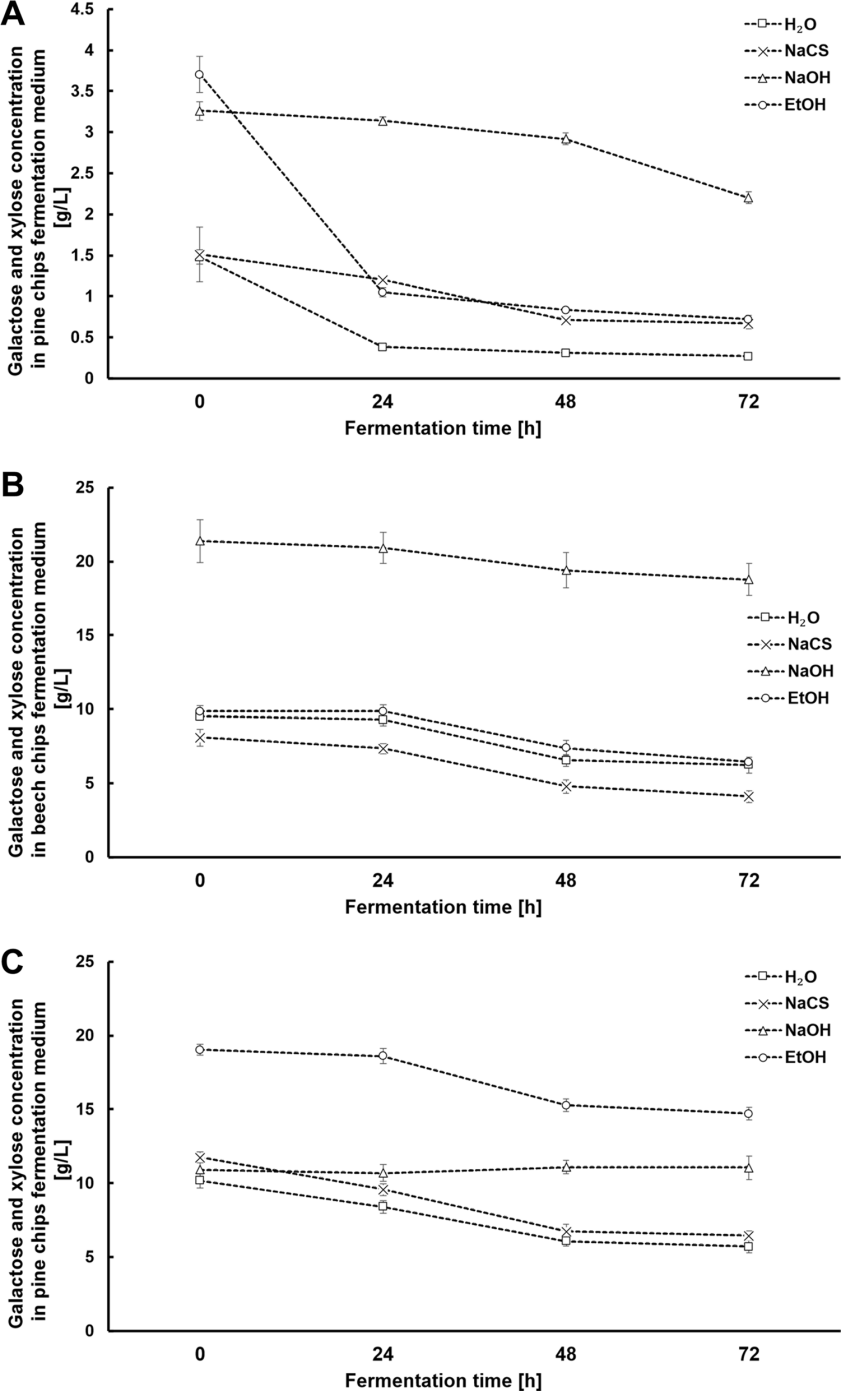
Changes in the concentration of galactose and xylose during alcoholic fermentation in hydrolysates obtained from pine chip (**A**), beech chip (**B**) and wheat straw (**C**) biomasses after high-pressure pretreatment using different solvents

**Fig.5:**
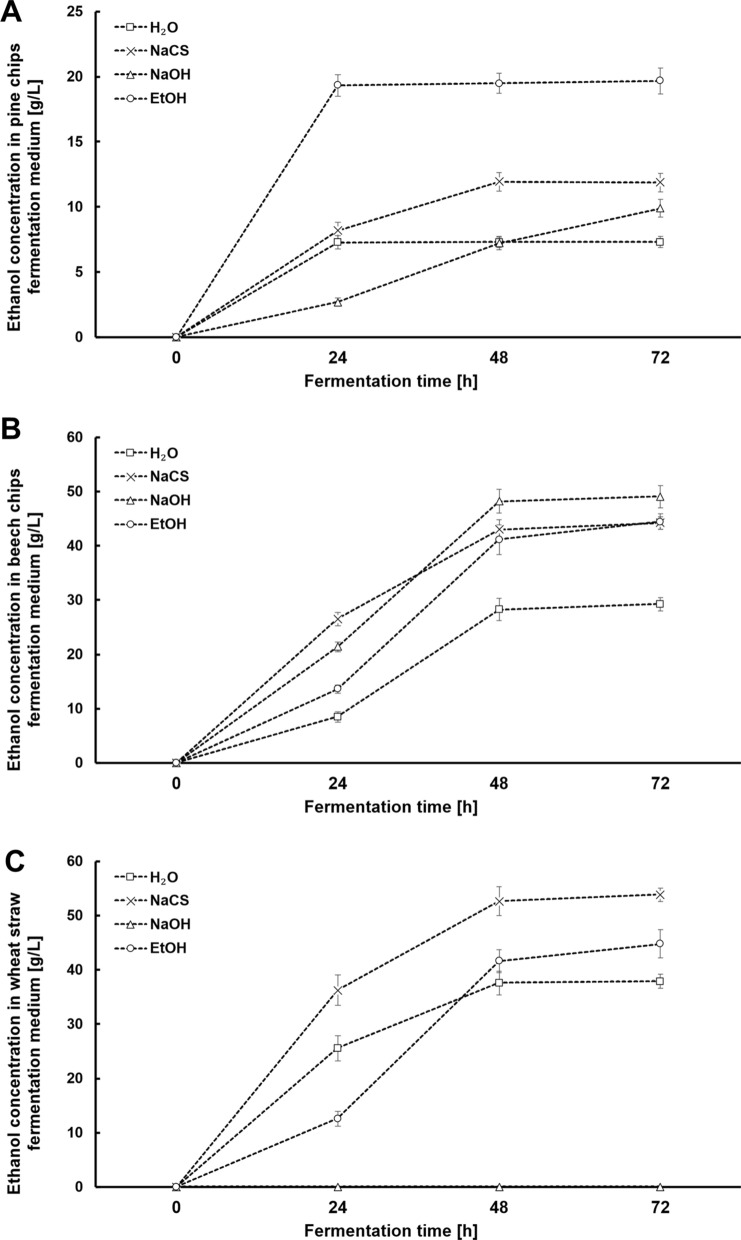
Changes in the concentration of ethanol during alcoholic fermentation in hydrolysates obtained from pine chip (**A**), beech chip (**B**) and wheat straw (**C**) biomasses after high-pressure pretreatment using different solvents

**Fig.6:**
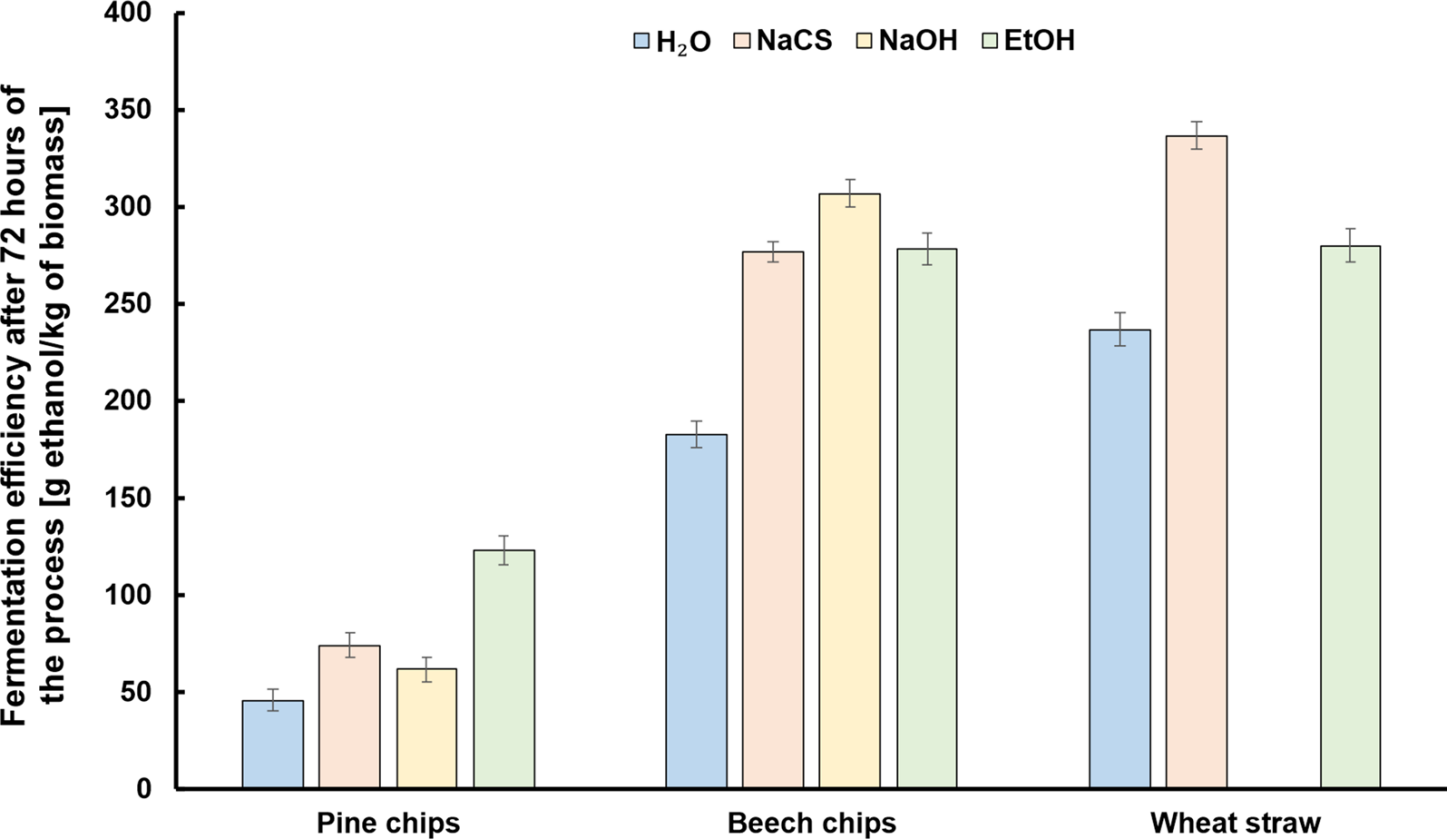
Effectiveness of alcoholic fermentation after 72 h of the process for different types of biomass

The characteristics of the course of fermentation of media for all variants of the experiment is presented in Figs. 3, 4 and 5. A high susceptibility of biomass to hydrolysis after high-pressure pretreatment using different solvents combined with a high content of biomass in the solution (160 g/L) enabled the generation of very good initial concentrations of glucose, which is a significant achievement of this study. For the pine chip media, the maximum concentration of glucose amounting to ca. 40 g/L was generated after pretreatment using EtOH. After pretreatment using NaCS, these media required 48 h, whilst media after biomass pretreatment using NaOH needed as many as 72 h for a full attenuation (Fig. 3A.). The obtained results indicate the need to supplement pine chip media with nutrients so as to fully attenuate glucose within a shorter time. A much higher initial concentration of glucose was obtained from the beech chip biomass after pretreatment using 40% *w*/*v* NaCS, 1% *w*/*v* NaOH or 60% *v*/*v* EtOH with an addition of 1% *v*/*v* H_2_SO_4_. It amounted to between 85 and 92 g/L (Fig. 3B.). Beech chip media reach full attenuation after 48 h of fermentation, which testifies to the presence of the sufficient amount of nutrients and the possibility of their use as a part of the HG technology. After high-pressure pretreatment using 40% *w*/*v* NaCS, the wheat straw medium was marked by the highest initial concentration of glucose at the level of ca. 110 g/L (Fig. 3C.). The generation of such a high initial concentration of glucose for cellulosic media (without concentrating the solution) is a significant achievement of the present study and indicates the desirability of using the presented ways of pretreatment of the raw materials in the production of media consistent with the HG technology. What is also important is an almost full attenuation of media generated from wheat straw within 48 h, which indicates a complementary composition of the media, guaranteeing a high rate of the bioconversion process (Fig. 3C). Fermentation media were also analysed for the presence of galactose and xylose as products of the residual degradation of hemicellulose (Fig. 4). In most media, we can observe only a slight lowering of the concentration of galactose and xylose testifying to the assimilation of galactose mainly between the 24th and 48th hour of fermentation (Fig. 4A, B, C). The kinetics of the production of ethanol in the obtained fermentation media is strongly correlated with the rate of the assimilation of fermentable sugars. A significant achievement of this stage of research was the generation of high final concentrations of ethanol using the particular lignocellulosic raw materials. In the pine chip media after pretreatment using EtOH, the maximum concentration of ethanol reached the level of ca. 20 g/L already in the 24th hour of fermentation (Fig. 5A). In similar tests covering an optimisation of the process of pretreatment of pine chip biomass in acidic conditions using surfactants, the maximum obtained concentration of ethanol was ca. 16 g/L, with the bioconversion of sugars at the level of ca. 63%, which, however, is markedly lower than that obtained in the research presented herein [40]. Problems with a complete bioconversion of sugars contained in the media from pine chip biomass were not observed when using high-pressure pretreatment in the environment of microwaves using 60% *v*/*v* EtOH with an addition of H_2_SO_4_ as a solvent (Fig. 5A). In view of a much higher concentration of glucose in beech chip and wheat straw media, the maximum concentration of ethanol in these media was observed only after as many as 48 h of fermentation. It amounted to ca. 49 g/L (Fig. 5B) for the beech chip medium after pretreatment using NaOH, and almost 54 g/L for the wheat straw medium after pretreatment using NaCS (Fig. 5C). The generation of such high concentrations of ethanol for hardwood media after various methods of pretreatment marks a progress in comparison to the research results published so far. Tests covering the use of the biological pretreatment of oakwood indicate the possibility of the generation of the final concentration of ethanol at the level of ca. 40 g/L [50]. In turn, as a part of the method of pretreatment of beech chips using a mixture of water and acetone as oxidising factors (acetone/water oxidised pretreatment), described as a method of production of highly concentrated cellulosic ethanol, only a slightly higher concentration of ethanol at the level of ca. 42 g/L was generated [51]. On the basis of a comparison of the presented results of our research with data from relevant literature, we may conclude that the achieved high concentration of ethanol in wheat straw medium after high-pressure microwave-assisted pretreatment using 40% *w*/*v* NaCS is an advance, an improvement of the technology of the production of second-generation ethanol. This is confirmed by the results of the use of wheat straw after dilute acid pretreatment on a pilot scale, reporting a possibility to generate an ethanol concentration at the maximum level of 30 g/L [52]. Even a simultaneous use of wheat straw after steam processing and wheat flour in the proportion 1:1 makes it possible to obtain an ethanol concentration at the maximum level of 50 g/L within 72 h of fermentation as a part of the technology of simultaneous saccharification and fermentation (SSF) [53]. A high concentration of ethanol obtained during our tests translates to a high efficiency of fermentation after 72 h of the process (Fig. 6). In the case of beech chips and wheat straw subjected to high-pressure pretreatment in the environment of microwaves using respectively solutions of NaOH and EtOH, more than 300 g of ethanol was obtained per kilogramme of biomass (Fig. 6). As a result of the presented pretreatment strategy, fermentation media marked by a high content of sugars assimilated by the *Saccharomyces cerevisiae* yeast and a high potential for cellulosic ethanol production in the HG technology were obtained from various types of lignocellulosic biomass.

## Conclusions

The achievement of the tests presented herein lies in the development of an effective method of the preparation of lignocellulosic material, involving a selection of process conditions for biomass pretreatment using a high-pressure microwave-assisted pretreatment assisted with the use of different solvents. An advantage of the presented method lies in the possibility of obtaining biomass which is highly susceptible to enzymatic hydrolysis and which thus makes it possible to obtain cellulosic hydrolysates with a high concentration of fermentable sugars meeting the criteria for HG media, without the need for concentration. The effectiveness of the presented method of pretreatment in the area of generating the desired structural changes in the biomass increasing its susceptibility to enzymatic hydrolysis has been confirmed by the FTIR, SEM and XRD analyses. Fermentation of lignocellulosic media in the HG standard, obtained as a result of the use of high-pressure microwave-assisted pretreatment and enzymatic hydrolysis, leads to the generation of high concentrations of ethanol at the level reaching 54 g/L (in the case of the use of wheat straw as a raw material), which allows to reduce the cost of the process of distillation. A translation of the achieved yield of ethanol into litres per tonne of biomass indicates a high effectiveness of the method presented, as confirmed by the 426 L of EtOH generated per tonne of wheat straw after pretreatment using NaCS and 388 L of EtOH generated for beech chips after pretreatment using NaOH.

## Materials and methods

### Raw materials

The lignocellulosic biomass used in our tests had the form of pine chips and beech chips from a sawmill (Wudzyń, Kujawsko-Pomorskie, Poland) and wheat straw from a farm (Radzicz, Kujawsko-Pomorskie, Poland). The biomass samples had a dry weight (DW) content of 95.05 ± 0.52%. Prior to the commencement of the study, all biomass samples were ground using a hammer mill and sieved through a sieve with an aperture size of 1 mm.

### Materials

The hydrotrope used for the pretreatment was sodium cumene sulfonate (NaCS, C_9_H_11_NaO_3_S) in the form of 40% w/v of Stepanate SCS 40 by Stepan Company (Northfield, Illinois, United States). Other reagents used for pretreatment, i.e. sulfuric acid, sodium hydroxide and ethanol were marked by their analytical grade purity and were manufactured by Merck (Darmstadt, Germany). Chromatographic analyses were conducted using a solution of sulfuric acid, methanol and acetic acid marked by HPLC grade purity and manufactured by Merck (Darmstadt, Germany). The standards of sugars (glucose, galactose and xylose), ethanol, glycerol and pretreatment by-products (5-HMF, furfural, 1,2-dihydrobenzene, 4-hydroxybenzoic acid, vanillic acid, sinapyl alcohol, guaiacol, syringaldehyde, furoic acid) used for chromatographic analyses were marked by their HPLC grade purity and originated from Merck (Darmstadt, Germany).

### Cellulolytic enzyme

Hydrolysis of cellulose was performed using Cellic^®^ CTec2 (Novozymes, Franklinton, NC, USA)—a preparation containing a complex of cellulolytic enzymes with the activity of 75 FPU/mL (Filter Paper Unit). The catalytic activity of the preparation was determined in consistence with the NREL methodology (NREL/TP-510-42,628). The preparation was used, as recommended by the manufacturer, at pH 5.5 and 50 °C.

### Yeast

The process of alcoholic fermentation was based on active dry alcohol yeast *S. cerevisiae,* Ethanol Red strain (Lesaffre Advanced Fermentations, Marcq-en-Baroeul, France). The yeast was added to the prepared cellulosic media in the form of yeast milk (1.25 ± 0.12 10^9^ CFU(colony-forming unit)/mL, viability 94.3 ± 08%) made by suspending 1 g of yeast preparation in 10 mL of sterile solution of 0.9% *w*/*v* NaCl followed by rehydration at 20 °C for 30 min (as recommended by the manufacturer) [54].

### Biomass pretreatment

Three types of lignocellulosic biomass, i.e. pine chips, beech chips and wheat straw were subjected to microwave-assisted pretreatment under controlled pressure conditions in the Mars 5 device by the CEM Corporation (Matthews, NC, USA) with water, 40% *w*/*v* NaCS, 1% *v*/*v* H_2_SO_4_, 1% *w*/*v* NaOH or 60% *v*/*v* ethanol (EtOH) with the addition of 1% *v*/*v* H_2_SO_4_ used as solvents. Preparations for microwave-assisted pretreatment were preceded by the weighing of 4.0 g DW of biomass in 10 repetitions to HP-500 Plus Teflon vessels and adding 80 mL of the appropriate solvent. The above was followed by pretreatment for 60 min under a constant pressure of 156 PSI, and the constant power of a microwave generator of 600 W. Isobaric conditions during the microwave-assisted pretreatment made it possible to generate the following temperatures for the particular solvents: 183 °C for water, 184 °C for 40% *w*/*v* NaCS, 181 °C for 1% *v*/*v* H_2_SO_4_, 181 °C for 1% *w*/*v* NaOH, and 164 °C for 60% *v*/*v* ethanol with the addition of 1% *v*/*v* H_2_SO_4_. After pretreatment, the solution was cooled to 50 °C and trickled under lowered pressure to separate the lignocellulosic biomass. After microwave-assisted pretreatment, the solution was analysed to determine the concentration of the by-products generated during the process. The lignocellulosic biomass was subsequently rinsed with 1000 mL of distilled water of 50 °C and dried to a dry mass, which was followed by the calculation of the biomass extractives on the basis of the difference between the biomass test portion before and after pretreatment. The acquired lignocellulosic biomass after pretreatment was used for an analysis of the lignocellulose composition, its susceptibility to enzymatic hydrolysis, degree of biomass crystallinity, efficiency of hydrolysis, FTIR and SEM analysis and the preparation of HG fermentation media.

### Enzymatic hydrolysis of cellulose

Lignocellulosic biomass of various types was subjected to enzymatic hydrolysis using the cellulolytic preparation Cellic^®^ CTec2 (Novozymes, Franklinton, NC, USA). For this purpose, 4 g DW of biomass was weighed and 25 mL 0.05 M of acetate buffer of pH 5.5 was added. The acquired solution was preliminarily incubated in a water bath of 50 °C for 5 min and the cellulolytic preparation was subsequently added in the amount of 16 FPU/g DW of biomass. The biomass solution was subjected to hydrolysis at 50 °C for 72 h with shaking at 70 rpm. The process of enzymatic hydrolysis was carried out in three repetitions for each type of biomass. After the completed process of enzymatic hydrolysis, the glucose concentration was determined on the basis of the HPLC method. Using the following formula, the efficiency of hydrolysis was calculated on the basis of the glucose concentration:

$${\text{Glucose yield }}\left[ {\text{\% }} \right] = { }\frac{{{\text{C}}_{{\text{Glu}}} }}{{1.111{ } \times {\text{ C}}_{{\text{Cel}}} { } \times {\text{ C}}_{{\text{Biom}}} }}{ } \times 100$$

where C_Glu_ is the glucose concentration (grams per litre) in the sample, C_Cel_—the content of the cellulose in the biomass (per gram of dry mass), and C_Biom_—the initial content of the biomass (grams per litre) [55].

### Cellulosic ethanol production

The process of alcoholic fermentation was carried out using the HG media containing 160 g DW of biomass per litre. Fermentation media were prepared in three repetitions using various types of biomass after pretreatment and different solvents. The preparation of fermentation media was commenced with the weighing of 8 g DW of biomass, adding 50 mL 0.05 M of acetate buffer of pH 5.5 and enzymatic hydrolysis using 16 FPU/g DW of biomass at 50 °C for 72 h with the shaking set at 70 rpm. After the completed enzymatic hydrolysis, the acquired hydrolysates were cooled to 30 °C and yeast in the form of a suspension was added in the amount of 0.8 mL (1.6 g/L). The flasks were subsequently closed with fermentation bungs containing glycerine and incubated at 35 °C. Before the commencement of the process of fermentation and after 24, 48 and 72 h of the process, the concentration of glucose, galactose and xylose, as well as ethanol, was monitored using the HPLC method. The effectiveness of the fermentation was determined on the basis of the final concentration of ethanol as expressed in grams of ethanol in one kilogramme of DW of biomass.

### Analytical methods

The biomass after pretreatment was analysed for lignocellulose composition using Fourier transform infrared spectroscopy (FTIR), scanning electron microscopy (SEM) and X-ray diffraction (XRD). The composition of lignocellulosic hydrolysates was determined using the HPLC-RID method, whilst solutions after pretreatment were analysed with the help of the HPLC-DAD method.

### Determination of cellulose, hemicellulose and lignins in biomass after pretreatment

The pretreated biomass was analysed for the content of cellulose, hemicellulose and lignins. The determination of lignocellulose components was carried out using Fibertec 8000^®^ by FOSS (Hilleroed, Denmark) in consistence with the international standards ISO13906:2008 and ISO16472:2006. The analysis involved determination of Neutral Detergent Fibre (NDF), Acid Detergent Fibre (ADF) and Acid Detergent Lignin (ADL) in the biomass with the subsequent calculation of the content of cellulose from the difference between the ADF and the ADL, and hemicellulose from the difference between the NDF and the ADF; lignin content was equal to the ADL [56].

### XRD analysis

An analysis of biomass crystallinity was performed with the help of an X-ray diffractometer Empyrean by PANalytical (Malvern, UK), using monochromatic CuKa radiation (1.54 Å) set at 40 kV, 20 mA. The goniometer scanned a 2θ range between 4.8479° and 90.9826° at a 0.0263° step size. The biomass crystallinity was determined using the empirical method in consistence with the following formula:

$$Biomass{ }\,crystallinity{ } = \frac{{I_{002} - I_{am} }}{{I_{002} }} \times 100{\text{\% }}$$

where I_002_ is a maximum intensity of the diffraction for crystalline and amorphous areas, and I_am_ is a minimum intensity of diffraction between the peaks 002 and 101, characteristic for amorphous areas [57].

### FTIR analysis

All the biomass samples were dried at 130 °C to a constant dry weight, ground and sieved through a sieve with an aperture size of 150 µm. Then a biomass sample of 2 mg was mixed with 200 mg KBr of spectroscopic grade purity and ground in an agate mortar for 5 min. The samples acquired in this way were pressed using a hydraulic press with the pressure of 2 tonnes for 5 min (Specac Ltd. Kent, England). The spectra were collected in the range from 4.000 to 400 cm^−1^ with a resolution of 4 cm^−1^ and 72 scans per sample on a Nicolet iS5 spectrometer (Thermo Fisher Scientific, Waltham, MA, USA).

### SEM analysis

The imaging of samples of lignocellulosic biomass of various types was performed using the scanning electron microscope Quanta 3D FEG by Fei (Hillsboro, OR, USA). The device worked in the conditions of a topographic contrast—detector ETD (SE) and accelerated voltage HV = 5 kV, in a state of high vacuum [44].

### HPLC-RID analysis

Determination of the concentration of glucose, galactose and xylose as well as ethanol was performed by high-performance liquid chromatography using chromatograph 1260 by Agilent Technologies^®^ (Palo Alto, CA, USA) equipped with the refractometric detector (HPLC-RID). For this purpose, 2 mL of hydrolysates and fermentation media were centrifuged at 12000 × g at 20 °C, diluted tenfold in 5 mM of H_2_SO_4_ and filtered through a membrane filter (PES) with an aperture size of 0.45 µm. The acquired samples were subjected to chromatographic analysis using the Hi-Plex H column (Agilent Technologies, Palo Alto, CA, USA) equipped with a guard column at 60 °C with the isocratic flow of the mobile phase (5 mM H_2_SO_4_) at 0.6 mL/min. Detection was carried out at 50 °C. Quantitative determinations were performed using the multilevel external standards method (ESTD) and the appropriate calibration curves. Since the Hi-Plex H column does not make it possible to effectively separate xylose and galactose peaks, the concentration of these sugars is given as a sum figure.

### HPLC–DAD analysis

The pretreated solutions were analysed for the presence of by-products. The analyses covered the determination of the concentration of 5-HMF, furfural, 1,2-dihydrobenzene, 4-hydroxybenzoic acid, vanillic acid, sinapyl alcohol, guaiacol, syringaldehyde and furoic acid. Samples of pretreated media were centrifuged at 12000 × g at 20 °C and filtered through a membrane filter (PES) with the aperture size of 0.22 µm. The determination was performed using liquid chromatograph 1260 by Agilent Technologies^®^ (Palo Alto, CA, USA) equipped with a diode detector (HPLC-DAD). Chromatographic separation was performed with the help of the column ZORBAX Eclipse Plus C18 (4.6 × 100 mm, 3.5 µm) (Agilent Technologies®, Palo Alto, CA, USA) using the mobile phase in the form of 0.3% acetic acid (70%) and methanol (30%) with the flow rate of 0.5 mL/min. in 30 ºC. Detection was carried out at two wavenumbers: 280 and 320 nm [58]. Quantitative calculation was carried out using the external standards method (ESTD) and the appropriate calibration curves.

### Statistical analysis

All laboratory analyses were performed in triplicate. Statistical analysis was carried out using the TIBCO Software Inc. Statistica ver. 13 (Palo Alto, CA, USA). An HSD Tukey’s test was applied at the significance level of α < 0.05.
